# Influence of the Infrapatellar Fat Pad Resection during Total Knee Arthroplasty: A Systematic Review and Meta-Analysis

**DOI:** 10.1371/journal.pone.0163515

**Published:** 2016-10-05

**Authors:** Chenyi Ye, Wei Zhang, Weigang Wu, Mingyuan Xu, Nwofor Samuel Nonso, Rongxin He

**Affiliations:** 1 Department of Orthopedic Surgery, The Second Affiliated Hospital, School of Medicine, Zhejiang University, Hangzhou, Zhejiang, 310009, China; 2 Department of Plastic Surgery, First Affiliated Hospital, School of Medicine, Zhejiang University, Hangzhou, 310009, China; Georgia Regents University, UNITED STATES

## Abstract

**Background:**

To enhance surgical exposure, resection of the infrapatellar fat pad (IPFP) is usually a routine procedure in total knee arthroplasty (TKA). However, there is conflicting evidence regarding whether IPFP resection during TKA impairs clinical outcome. We performed a systematic review and meta-analysis to clarify the influence of IPFP resection on primary TKA.

**Methods:**

Embase, PubMed, and the Cochrane Library were systematically searched up to August 2016 to identify relevant studies. All clinical studies comparing IPFP resection (IPFP-R) and IPFP preservation (IPFP-P) in patients undergoing primary TKA were obtained. The meta-analysis was performed with Revman 5.3 and STATA 12.0 software. The weighted mean was estimated by using random effects (RE) models with 95% CIs, heterogeneity was assessed using the *H* statistic and the inconsistency index (*I*^*2*^).

**Results:**

Seven studies involving 2,734 patients (3,258 knees) were included. IPFP resection trended to increase the incidence of postoperative anterior knee pain within 2 months postoperatively, compared with patients in whom the IPFP was preserved (odds ratio [OR]s 2.12[0.95, 4.73], *p* = 0.07). An increased incidence of anterior knee pain was observed in the IPFP resection group > 12 months postoperatively, but the difference was not significant (OR, 3.69 [0.81, 16.82], *p* = 0.07). In addition, a trend towards more shortening of the patellar tendon was also observed in the IPFP-R group. No significant results were found regarding postoperative knee function.

**Conclusion:**

These results suggest that preserving the IPFP may be superior to IPFP resection in patients undergoing primary TKA, due to the relatively lower rate of anterior knee pain after short-term follow-up.

## Introduction

Resecting the infrapatellar fat pad (IPFP, retropatellar fat pad, or Hoffa’s fat pad) is usually a routine procedure during primary total knee arthroplasty (TKA) to enhance surgical exposure or prevent interposition during prosthesis implantation [1]. The 2004 National Joint Registry report for England and Wales showed that the IPFP was totally or partially removed in 86% of primary TKAs and only 14% of IPFPs were preserved [2]. However, there is conflicting evidence on outcomes after primary TKA regarding IPFP resection [3, 4].

The IPFP is a fatty mass lying beneath the patellar ligament, which contains transverse infrapatellar arteries that supply the patella and the anterior part of the knee joint [5, 6]. Resecting the IPFP interrupts the peripatellar artery ring, theoretically resulting in patellar avascular necrosis and fracture [7–10]. Furthermore, the IPFP is a cushion between the anterior tibial plateau and patellar tendon; its removal can result in anterior impingement, patella baja, anterior knee pain, and reduced flexion after TK [5, 11–16]. A cadaveric study by Bohnsack et al. showed that resecting the IPFP may decrease medial translation and external rotation of the patella, as well as shorten the patellar tendon [17].

On the other hand, some clinical studies have reported no functional differences [14, 18, 19], anterior knee pain [20], or shortening of the patella tendon [14, 18, 19] between patients who received IPFP resection (IPFP-R) and IPFP preservation (IPFP-P) during TKA. In addition, many surgeons resect the IPFP for adequate surgical exposure.

The overall effects of resecting the IPFP on TKA outcomes are unclear. Moreover, a number of comparative studies have considered this issue recently. Therefore, it is essential to perform a systematic review and meta-analysis for clarification.

## Methods

This study was performed in accordance with the Preferred Reporting Items for Systematic Reviews and Meta-Analyses guidelines [21] and the Cochrane Handbook for Systematic Reviews of Interventions (ver. 5.0.2).

### Literature Search

Three independent investigators (CY, WZ, and WW) searched electronic databases (Embase, PubMed, and the Cochrane Library) with no language restrictions through August 2016. The following keywords or corresponding Medical Subject Headings were used: “infrapatella fat pad” or “IPFP” or “retropatellar fat pad” or “Hoffa’s fat pad” and “total knee arthroplasty” or “total knee replacement” or “TKA” or “TKR” or “total joint replacement” or “total joint arthroplasty” or “TJA” or “TJR”. Details of the search strategy are provided in S1 and S2 Texts. Reference lists were also searched manually for additional studies. The Clinical Trial Registry, the National Institutes of Health, and the Current Controlled Trials databases were also searched for unpublished trials and for those in progress.

### Inclusion and Exclusion Criteria

Two reviewers (CY and WZ) independently screened manuscript titles and abstracts, and implemented the following inclusion criteria: (1) full text had to be readily available; (2) comparative studies directly comparing IPFP-R and IPFP-P for primary TKA; (3) studies that enrolled individuals aged ≥18 years; and (4) studies that provided original postoperative data.

The following exclusion criteria were used in this study: (1) articles that did not satisfy the inclusion criteria; (2) animal studies, letters, reviews, abstracts, conference proceedings, case reports, or systematic reviews; and (3) studies that did not provide sufficient clinical measurements of function, pain, patellar tendon length, or patellar position.

### Data Extraction

Three independent reviewers (CY, WZ, and WW) obtained relevant data and assessed the accuracy. The following information was extracted from each study: first author’s name, year of publication, country, study design, patient demographics, results of postoperative pain, function, and results of the radiological evaluation for patellar tendon shortening and patellar position. Contact with corresponding authors was also attempted to verify the accuracy of the data and obtain further data for the analysis.

### Quality Assessment

Three investigators (CY, WZ, and WW) independently assessed the methodological quality of each study on a 12-item scale [22]. The 12-item scale contained the following: adequate randomization, allocation concealed, care provider blinded, patient blinded, outcome assessor blinded, acceptable dropout rate, intention-to-treat analysis, similar baseline, similar or avoided cofactor, similar timing, patient compliance, and avoided selective reporting. Any disagreements were evaluated using the kappa test, and consensus was reached by discussion with the corresponding author (RH). The articles with “yes” answers to items ≥ 7 were classified as high quality, and studies with “yes” answers to items < 4 were low quality [22].

### Statistical Analysis

Revman software (ver. 5.3; The Nordic Cochrane Centre, Copenhagen, Denmark) and STATA 12.0 (Stata Corp, College Station, Texas) were used to pool the data. A p-value ≤ 0.05 was considered significant. Relative risk and 95% confidence intervals (CIs) were used for dichotomous outcomes. For continuous data, standardized mean difference (SMD) or weighted mean difference was calculated with the 95% CI as a summary statistic. The inconsistency index (*I*^*2*^),and the *H* statistic were used to assess heterogeneity between the studies [23, 24]. *I*^*2*^ values of 25, 50, and 75% were considered low, medium, and high heterogeneity, respectively [25]. *H*^*2*^and confidence intervals for *I*^*2*^ calculated using STATA software were provided whenever applicable [26, 27]. For any heterogeneity, the random-effects (RE) models, which were reported to be more conservative and provide better estimates with wider confidence intervals than fixed effect (FE) models [28, 29], were used to estimate the weighted mean. Subgroup analyses were conducted according to the follow-up time.

## Results

### Literature Search

**Fig 1:**
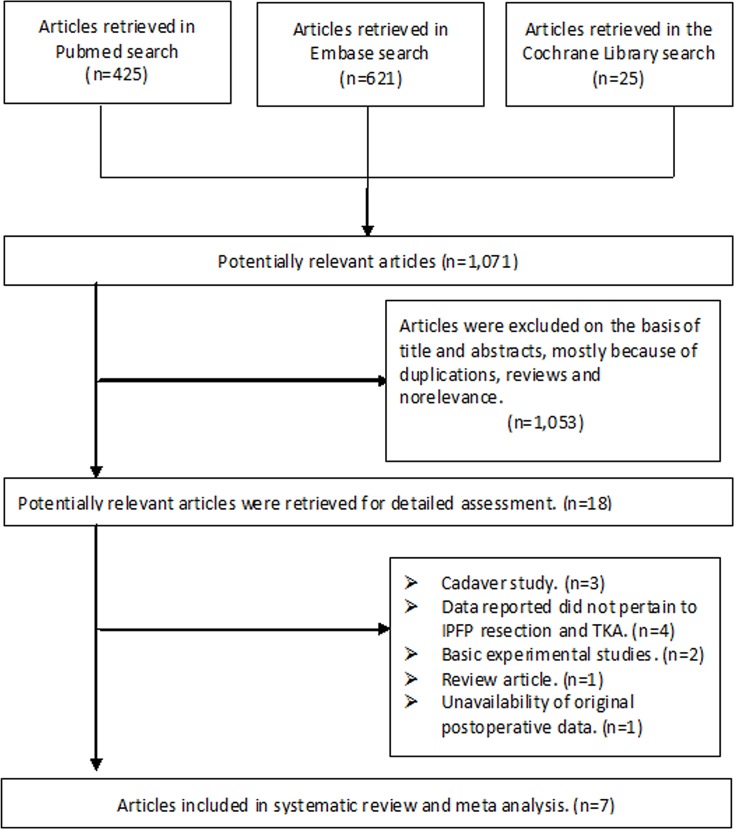
A PRISMA flowchart illustrated the selection of studies included in our systematic review.

In total, 1,071 candidate publications were identified; however, 1,053 were excluded due to duplications, irrelevance, or because they were not comparative studies between IPFP-R and IPFP-P. After assessing the 18 potentially relevant articles, seven studies (three random control trials [RCTs], two cohort studies, and two case-control studies) involving 2,734 patients (3,258 knees) met the inclusion criteria [1, 14, 18–20, 30, 31]. The primary reasons for exclusion were that three were cadaveric studies [5, 8, 32], and two were basic experimental studies [8, 33]. Four studies failed to relate the data to IPFP resection and TKA [6, 34–36], one study was excluded because of the unavailability of original usable data [37], and one study was excluded because it was a review article [38]. The details of study selection are presented in Fig 1. The weighted kappa for the agreement on eligibility between the reviewers was 0.87 (95% CI, 0.84–0.92).

### Study Characteristics and Methodological Quality of the Studies

The characteristics and quality assessment results of the seven studies are summarized in Table 1.

**Table 1:** Methodological quality of the included studies based on the 12-items scoring system.

Study	Randomized	Allocation	Patient	Care provider	Outcome assessor	Acceptable	ITT	Avoided selective	Similar	Similar or	Patient	Similar	Quality^e^
	adequately^a^	concealed	blinded	blinded	blinded	drop-out rate^b^	Analysis^c^	reporting	baseline	avoided cofactor	compliance^d^	timing	
Tanaka [1], 2003	Yes	Yes	Unclear	Unclear	Unclear	Yes	Yes	No	Yes	Yes	Yes	Yes	High
Maculé, 2005[19]	Yes	Yes	Unclear	Unclear	Unclear	Yes	Yes	No	No	Yes	Yes	Yes	High
Lemon, 2007[30]	No	No	No	No	No	Yes	Yes	Yes	No	Yes	Yes	No	Moderate
Meneghini, 2007[14]	No	No	No	No	No	Yes	Yes	No	Yes	Yes	Yes	Yes	Moderate
Moverley, 2014[31]	No	No	No	No	No	Yes	Yes	No	Yes	Yes	Yes	Yes	Moderate
Pinsornsak, 2014[18]	Yes	Yes	Unclear	Unclear	Unclear	Yes	Yes	Yes	Yes	Yes	Yes	Yes	High
Seo, 2015[20]	No	No	No	No	No	Yes	Yes	No	No	Yes	Yes	Yes	Moderate

^a^ Only if the method of sequence made was explicitly introduced could get a ‘‘Yes”; sequence generated by ‘‘Dates of Admission” or ‘‘Patients Number” receive a ‘‘No”.

^b^ Drop-out rate <20% could get a ‘‘Yes”, otherwise ‘‘No”.

^c^ ITT = intention-to-treat, only if all randomised participants were analysed in the group they were allocated to could receive a ‘‘Yes”.

^d^ More than 75% patients wore respective devices for at least 3 weeks means ‘‘Yes”, otherwise ‘‘No”.

^e^ ‘‘Yes” items more than 7 means ‘‘High”; more than 4 but no more than 7 means ‘‘Moderate”; no more than 4 means ‘‘Low”.

Of them, three studies were RCTs, two were cohort studies, and two were case-control studies.

Participants ranged in age from 33 to 96 years, with a mean age of 70.64 years. A total of 2,734 patients (3,258 knees) were included (916 males and 1,818 females). The indications for TKA were osteoarthritis (OA) in three studies [19, 20, 30], rheumatoid arthritis (RA) in one study [1], 97.9% OA + 1.3% RA + 0.8% osteonecrosis (ON) in one study [14], 93.5% OA+6.5% RA in one study [18], and unclear in one study [31]. Patella resurfacing was done in all patients in three studies [1, 14, 20]. Two studies did not resurface the patella [19, 30], and two studies included both patients with/without patella resurfacing [18, 31]. The follow-up period varied from 1 month to 5 years and no loss of follow-up > 20% was observed in any of the studies (Table 2). Three studies that explicitly used randomization and allocation concealment were considered high quality, and the other four studies were of moderate quality (Table 3).

**Table 2:** Characteristics of subjects in eligible studies.

Studies	Study	Mean Age	No. of knees		No. of patients		Male	Indication	Patella	Follow-up	Loss to	Ant knee	Patellar	Function	Prosthesis
	design	(years)	IPFP-R	IPFP-P	IPFP-R	IPFP-P	/female	TKA	resurfacing	(months)	follow up	pain	tendon length		
Tanaka [1], 2003	RCT	54.1	54	53	40	40	20/60	RA	Y	33	0	↑	↓	↓	Nex-gen PS
Maculé, 2005[19]	RCT	71.60	34	34	34	34	10/58	OA	N	6	0	↓	=	=	Profix PCL preserving
Lemon, 2007[30]	Cohort	72.60	35	38	35	38	48/25	OA	N	36	0	NA	↓	NA	Profix
Meneghini, 2007[14]	Case-control	69.40	285	770	169	451	281/439	OA+RA+ON	Y	61.2	38	↑	=	=	AGC/ Legacy LPS
Moverley, 2014[31]	Cohort	71.90	1231	186	NA	NA	518/883	NA	Y/N	12	0	↑	NA	↓	NA
Pinsornsak, 2014[18]	RCT	67.70	45	45	45	45	5/72	OA+RA	Y/N	12	13	↑	=	=	MIS Quad-Sparing^TM^
Seo, 2015[20]	Case-control	69.0	201	247	140	162	34/268	OA	Y	< 1	0	=	NA	NA	LOSPA PS

IPFP-R: infrapatella fat pad resection; IPFP-P: infrapatella fat pad preservation. OA: Osteoarthritis; RA: Rheumatoid arthritis; ON: Osteonecrosis; TKA: total knee arthroplasty; PS: posterior stabilized; PCL: posterior cruciate ligament; Ant knee pain: anterior knee pain. Y: with patella resurfacing; N: without patella resurfacing.

**Table 3:** Bias screening using the Cochrane scale collaboration.

	Cochrane scale collaboration					
Study	Selection bias	Performance bias	Detection bias	Attrition bias	Reporting bias	Other bias
Tanaka, 2003[1]	High risk	High risk	High risk	Low risk	Low risk	Low risk
Maculé, 2005[19]	High risk	High risk	High risk	Low risk	High risk	Low risk
Lemon, 2007[31]	High risk	High risk	High risk	Low risk	Low risk	Low risk
Meneghini, 2007[14]	High risk	High risk	High risk	Low risk	Low risk	Low risk
Moverley, 2014[32]	High risk	High risk	High risk	Low risk	Low risk	Low risk
Pinsornsak, 2014[18]	High risk	High risk	High risk	Low risk	Low risk	Low risk
Seo, 2015[20]	High risk	High risk	High risk	Low risk	Low risk	Low risk

### IPFP-R Trended to Decrease Anterior Knee Pain

Summary of results between IPFP resection and IPFP preservation groups were shown in Table 4.

**Table 4:** Summary of results between IPFP resection and IPFP preservation groups.

Outcomes	Studies	Participants	Mean [95% CI]	*I*^*2*^ for heterogeneity	*P* value
1. Pain—OR					
1~2 months postoperatively	3	265	2.23 [1.06, 4.69]	7%	*P*<0.05
>12 months postoperatively	3	1252	3.69 [0.81, 16.82]	74%	*P =* 0.07
2. LT shortening—SMD					
1~2 months postoperatively	2	174	-0.15 [-0.45, 0.15]	0%	*P* = 0.33
6~12 months postoperatively	2	141	0.37 [-0.54, 1.28]	86%	*P* = 0.42
>24 months postoperatively	2	179	1.00 [0.41, 1.60]	72%	*P*<0.05

IPFP: infrapatellar fat pad; OR: Odds Ratio; LT: length of patellar tendon; SMD: standardized mean difference.

**Fig 2:**
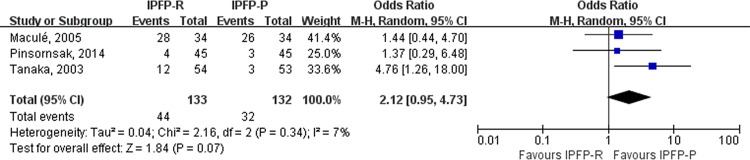
Forest plot shows that IPFP resection trended to increase the incidence of anterior knee pain within 2 months postoperatively comparing with the IPFP preservation group.

The incidence of anterior knee pain was higher in the IPFP-R group compared with that in the IPFP-P group (odds ratio [OR], 2.12; 95% CI, 0.95–4.73, *p* = 0.07; *p* for heterogeneity < 0.05, *I*^*2*^ = 7% [0%, 90%], *H* = 1.0 [1.0, 3.2], *H*^*2*^ = 1) within 2 months postoperatively. A similar trend was observed more than 12 months after surgery, although it was not significant (OR, 3.69; 95% CI, 0.81–16.82; *p* = 0.07). The results of *H* statistic and *I*^*2*^indicated obvious heterogeneity (*p* for heterogeneity < 0.05, *I*^*2*^ = 74% [14%, 92%]; *H* = 2.0 [1.1, 3.6], *H*^*2*^ = 4) [27]. However, due to the limited number of studies, we were unable to conduct a sensitivity analysis [Fig 2].

### IPFP-R Shortens the Patellar Tendon

**Fig 3:**
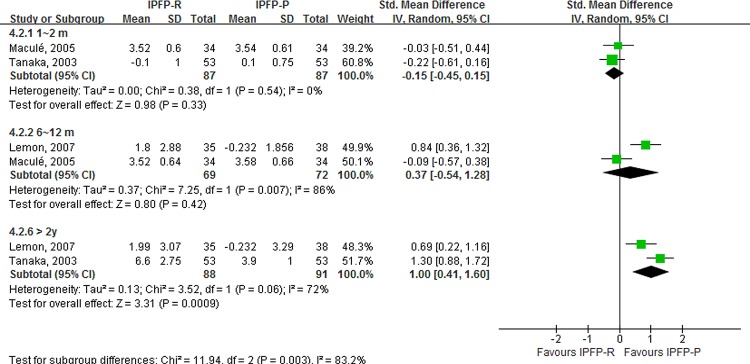
Forest plot about postoperative patellar tendon length shortening.

We found a trend toward more patellar tendon shortening in the IPFP-R group. The results of meta- analysis showed less patellar tendon shortening in the IPFP-R 1–2 months postoperatively than that in the IPFP group (SMD, −0.15; 95% CI, −0.45–0.15; *p* for heterogeneity = 0.54, I^2^ = 0%). The results of 6–12 months and >2 year postoperatively showed that IPFP-R decreased the patellar tendon (SMD, 0.37; 95% CI, −0.54–1.28; *p* = 0.42; *p* for heterogeneity < 0.05, I^2^ = 87%) and (SMD, 1.00; 95% CI, 0.41–1.60; *p* < 0.05; *p* for heterogeneity = 0.06, I^2^ = 72%), respectively. However, due to the limited number of studies (two studies per subgroup), *H* statistic and sensitivity analysis were not able to be conducted; thus, the role of heterogeneity should be considered when interpreting these results [39] [Fig 3].

### Range of Motion and Function

Two studies demonstrated that resecting the IPFP significantly decreased postoperative knee flexion and function [1, 31], whereas three studies showed no difference between IPFP-R and IPFP-P regarding range of motion (ROM) and function [14, 18, 19]. Due to the lack of data presented in the form of mean ± standard deviation, no statistical analysis was possible.

Four studies [1, 14, 18, 19] reported the results of knee flexion, and two studies [14, 31] reported postoperative function scores. Tanaka et al. studied inferior synovectomy with/without IPFP resection in patients with RA undergoing TKA and showed that fat pad resection increased the number of patients with weakness in the quadriceps muscles, and restricted knee motion 28–38 months after surgery [1]. However, in a case control study of 1,055 patients with more than 5 years of follow up, Meneghini et al. demonstrated no difference between IPFP-R and IPFP-P regarding Knee Society Score (KSS), KSS function score, or ROM [14]. Pinsornsak et al. reported 90 patients (93.5% OA + 6.5% RA) undergoing TKA and found no differences between IPFP-R and IPFP-P regarding knee flexion at 6 weeks, and 3, 6, and 12 months after surgery [18]. Maculé et al. also observed no differences in ROM between the IPFP-R and IPFP-P groups of patients with OA [19].

### Operation Time

One of the seven studies [20] showed that operation time was longer in the IPFP-P group than that in the IPFP-R group (70 ± 10.8 vs. 64 ± 10.0 min, *p* < 0.001).

## Discussion

To date, this is the first meta-analysis to evaluate the effect of rescting the IPFP in TKA, although two reviews involved it have been published [3, 4]. The present meta-analysis includes seven studies involving 2,734 patients (3,258 knees) [1, 14, 18–20, 30, 31]. Our results show a trend towards greater shortening of the patellar tendon and an increased incidence of anterior knee pain in patients who underwent IPFP resection during TKA. No differences were found regarding postoperative ROM or function.

The IPFP is a fatty mass lying beneath the patellar ligament, which contains transverse infrapatellar arteries that supply the patella and the anterior part of the knee joint [5, 6]. Resecting the IPFP can result in patellar avascular necrosis and fracture [7–10]. Other functions of the IPFP include enhancing stability and reducing friction in the knee joint [40]. As a cushion between the anterior tibial plateau and patellar tendon, the IPFP also protects the patellar tendon [5, 11–16, 40].

Postoperative anterior knee pain is a common complication after TKA [41]. Although it was difficult to differentiate the anterior knee pain caused by various factors, our study showed a trend towards an increased incidence of anterior knee pain after TKA in the IPFP-R group. The reported reasons for anterior knee pain include incomplete IPFP (caused by surgeries or trauma), postoperative scarring, anterior impingement, patella baja, and malalignment of the patella [5, 11–16]. Over-secretion of inflammatory mediators is also recognized as a factor [42]. The IPFP is a special type of connective tissue rich in fat cells, fibroblasts, and adipose-derived stem cells, which secrete a large number of anti-inflammatory factors, such as leptin and adiponectin, that further increase the proteoglycan synthesis and decrease secretion of tumor necrosis factor alpha and interleukin-1 in RA [42, 43].

Shortening of the patellar tendon is one of the most common complications after TKA. We found a trend towards greater shortening of the patellar tendon in the IPFP-R group than that in the IPFP-P group. Numerous factors are responsible for the shortening, including peripatellar soft tissue scar contraction, interruption of the peripatellar arterial ring, and weakness of the quadriceps muscles [44–47]. The cavity that forms after resecting the IPFP is filled with fibrous tissue; preserving the IPFP reduces the formation of a scar and contraction. Based on the previous studies, preserving the IPFP not only reduces or avoids interrupting the patellar tendon blood supply but also reduces the incidence of patellar tendon scarring and contraction. Furthermore, shortening the patellar tendon is also responsible for postoperative anterior knee pain and malalignment of the patella. A cadaveric study by Bohnsack et al. showed that resecting the IPFP may not only result in shortening of the patellar tendon but also decrease medial translation and external rotation of the patella [17].

Beeck et al. [3] conducted a systematic review in 2013 and demonstrated no differences in anterior knee pain, function, or ROM in patients with OA patients. They reported a trend towards decreased function and more discomfort in patients with RA. In this study, the indications for TKA were OA in three studies [19, 20, 30], RA in one study [1], 97.9% OA + 1.3% RA + 0.8% ON in one study [14], 93.5% OA + 6.5% RA in one study [18], and unclear in one study [31]. The OA cases contributed 92.53% of the study population for studies with clear indications [1, 14, 18–20, 30]. There is evidence that the IPFP preservation is an additional source of postoperative anterior knee pain in patients with RA [48–51]. However, the only study about RA patients included by Tanaka et.al. showed that there was a trend towards more discomfort and a decrease in function in RA patients [1, 3]. Based on the results of our study and the previously reported systematic review [3], the IPFP should be preserved whenever possible in patients with OA; however, due to the limited studies about the IPFP-R in RA patients, whether IPFP should be resected in patients with RA is still of question. Future RCTs focusing on individuals with different indications (*e*.*g*., OA, RA, and ON) for TKA are requested.

In the other systematic review conducted in 2016, White et al reported moderate evidence about increased knee pain with IPFP-R, while no difference in ROM was found between IPFP-R and IPFP-P, which was quite similar with the results of the present study. However, conflicting evidence was found regarding the patellar tendon length post-TKA [4]. By conducting further statistical analysis, we observed a trend towards greater shortening of the patellar tendon. However, as justified by White et al [4] and Lemon et al. [30], the use of Insall-Salvati ratio (ISR) might be underpowered to differentiate between a change on LT and a change in the joint height as the cause of the change in patella height. Thus, more RCTs with large scale reporting the change of LT using other measurements are required.

Our study had several strengths. Only two previous review article had assessed outcomes of primary TKA with IPFP resection [3, 4]. However, due to the limited number of available studies on this issue, those authors did not conduct a meta-analysis to further clarify the influence of IPFP resection on primary TKA. A number of comparative studies have addressed this issue recently. In addition, as described in the “Limitation” of the recently published systematic review, the majority of patients in their study were from a single retrospective study, which may decrease the robustness of the their conclusions. The present study is the first meta-analysis to investigate whether the IPFP should be removed routinely during TKA. We only included recently published studies (2000–2016) with comparable surgical techniques. Moreover, this study was a relatively comprehensive, up-to-date summary of data on the topic. We also conducted a subgroup analysis based on the postoperative period. Compared with the original studies, the larger number of cases and participants increased the detection rate of significant associations and provided more precise estimates of their effects.

This study also had several limitations. We included case-control and cohort studies to provide an updated overview of the clinical consequences of IPFP resection during TKA, which decreased the robustness of the conclusions. Second, obvious heterogeneity was observed regarding the anterior knee pain in more than 12 months postoperatively and the change in patellar ligament length. Although a subgroup analysis according to the follow-up time was conducted, we were unable to conduct a sensitivity analysis due to the limited number of included studies. Also, because of the number of included studies, publication bias tests were underpowered to be conducted. Together with the highly varied outcome measures, number of participants, and short-term follow-up periods, the role of heterogeneity and publication bias should be considered when interpreting these results. Nevertheless, our study provides useful insight into the effect of resecting the fat pad on TKA outcomes. Future RCTs should focus on individuals with different indications (*e*.*g*., OA, RA and ON) for TKA, and individuals who underwent complete or partial IFPF resection should also be included.

## Conclusion

In conclusion, our systematic review and meta-analysis indicates that preserving the IPFP may be superior to resecting the IPFP during primary TKA based on the relatively decreased incidence of anterior knee pain and a trend of less shortening in the patellar tendon.

## Supporting Information

S1 File RevMan data. (RM5)

S1 PRISMA Checklist PRISMA 2009 Checklist. (DOC)

S1 Table Search strategy for PubMed. (DOCX)

S1 Text Search strategy for Embase. (DOCX)

S2 Text Search strategy for Cochrane Library. (DOC)
