# Supplementary material for: Influence of the Infrapatellar Fat Pad Resection during Total Knee Arthroplasty: A Systematic Review and Meta-Analysis
Source: PLoS One. 2016 Oct 5;11(10):e0163515. doi: 10.1371/journal.pone.0163515 (PMC5051736; doi:10.1371/journal.pone.0163515)

S1 Table. Search strategy for PubMed

| **Search strategies in Pubmed: #3** |
| --- |
| #3  Search (((((((fat pad[Title/Abstract]) OR infrapatella fat pad[Title/Abstract]) OR retropatellar fat pad[Title/Abstract]) OR Hoffa’s fat pad[Title/Abstract]) OR IFP[Title/Abstract]) OR IPFP[Title/Abstract])) AND (((((((((knee[Title/Abstract]) OR total knee arthroplasty[Title/Abstract]) OR total knee replacement[Title/Abstract]) OR TKA[Title/Abstract]) OR TKR[Title/Abstract]) OR total joint replacement[Title/Abstract]) OR total joint arthroplasty[Title/Abstract]) OR TJA[Title/Abstract]) OR TJR[Title/Abstract])  #2  Search ((((((((knee[Title/Abstract]) OR total knee arthroplasty[Title/Abstract]) OR total knee replacement[Title/Abstract]) OR TKA[Title/Abstract]) OR TKR[Title/Abstract]) OR total joint replacement[Title/Abstract]) OR total joint arthroplasty[Title/Abstract]) OR TJA[Title/Abstract]) OR TJR[Title/Abstract]  #1  Search (((((fat pad[Title/Abstract]) OR infrapatella fat pad[Title/Abstract]) OR retropatellar fat pad[Title/Abstract]) OR Hoffa’s fat pad[Title/Abstract]) OR IFP[Title/Abstract]) OR IPFP[Title/Abstract] |
|  |
|  |


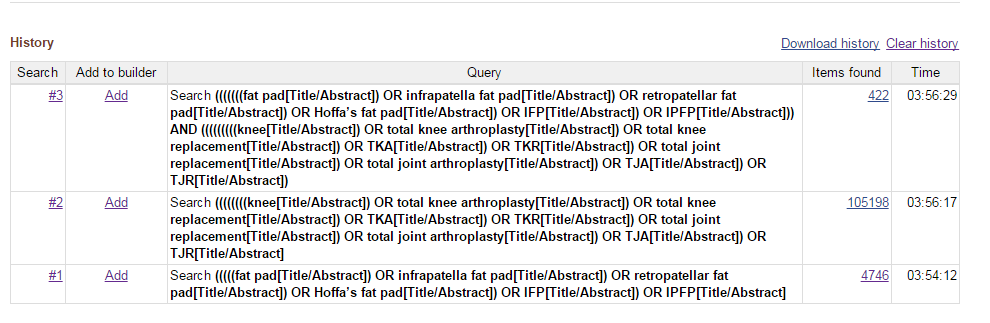

Supplement: S1 Table — (DOCX) [file pone.0163515.s003.docx]
