# Supplementary material for: Influence of the Infrapatellar Fat Pad Resection during Total Knee Arthroplasty: A Systematic Review and Meta-Analysis
Source: PLoS One. 2016 Oct 5;11(10):e0163515. doi: 10.1371/journal.pone.0163515 (PMC5051736; doi:10.1371/journal.pone.0163515)

**S1 Text. Search strategy for Embase**

**Embase search strategy for Embase**

**#3**  **#1** combined **#2**

**#2** 'knee' OR 'total knee arthroplasty' OR 'total knee replacement' OR 'TKA' OR 'TKR' OR 'total joint replacement' OR 'TKR' OR 'total joint arthroplasty' OR 'TJA' OR 'TJR'

**#1** 'fat pad' OR 'infrapatella fat pad'/exp OR 'infrapatella fat pad' OR 'retropatellar fat pad' OR 'retropatellar fat pad'/exp OR 'Hoffas fat pad' OR 'Hoffas fat pad'/exp OR 'IPFP' OR 'IPFP'/exp OR 'IFP' OR 'IFP'/exp


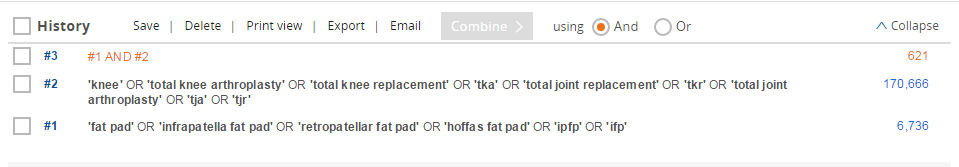

Supplement: S1 Text — (DOCX) [file pone.0163515.s004.docx]
