# Supplementary material for: Influence of the Infrapatellar Fat Pad Resection during Total Knee Arthroplasty: A Systematic Review and Meta-Analysis
Source: PLoS One. 2016 Oct 5;11(10):e0163515. doi: 10.1371/journal.pone.0163515 (PMC5051736; doi:10.1371/journal.pone.0163515)

**S2 Text. Search strategy for Cochrane Library**

**Search strategies in the Cochrane Library:**

#1

"fat pad":ti,ab,kw or infrapatella fat pad:ti,ab,kw or "retropatellar fat pad":ti,ab,kw or Hoffa’s fat pad:ti,ab,kw or IPFP:ti,ab,kw (Word variations have been searched)

#2

"total knee arthroplasty":ti,ab,kw or "total knee replacement":ti,ab,kw or "TKA":ti,ab,kw or "TKR":ti,ab,kw or "knee":ti,ab,kw (Word variations have been searched)

#3

#1 and #2


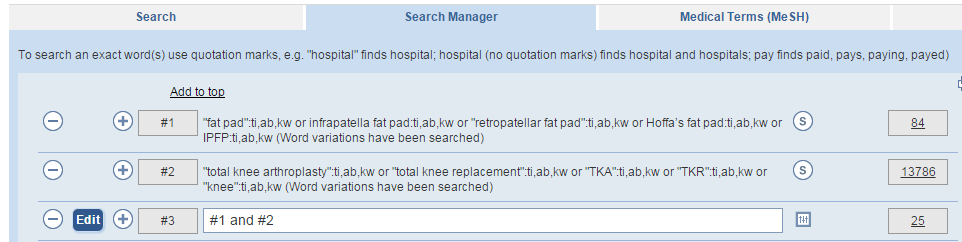

Supplement: S2 Text — (DOC) [file pone.0163515.s005.doc]
